# Anti-inflammatory activities of novel heat shock protein 90 isoform selective inhibitors in BV-2 microglial cells

**DOI:** 10.3389/fmolb.2024.1405339

**Published:** 2024-05-02

**Authors:** Amanda G. Smith, Valentin M. Kliebe, Sanket Mishra, Ryan P. McCall, Megan M. Irvine, Brian S. J. Blagg, Wei Lei

**Affiliations:** ^1^ Department of Pharmaceutical and Administrative Sciences, Presbyterian College School of Pharmacy, Clinton, SC, United States; ^2^ Department of Biology, Presbyterian College, Clinton, SC, United States; ^3^ Department of Chemistry and Biochemistry, University of Notre Dame College of Science, Notre Dame, IN, United States; ^4^ Department of Pharmaceutical and Graduate Life Sciences, Manchester University Fort Wayne, Fort Wayne, IN, United States

**Keywords:** heat shock protein 90, microglial cell, inflammation, pain, LPS

## Abstract

Heat shock protein 90 (Hsp90) is a family of chaperone proteins that consists of four isoforms: Hsp90α, Hsp90β, glucose-regulated protein 94 (Grp94), and tumor necrosis factor type 1 receptor-associated protein (TRAP1). They are involved in modulating the folding, maturation, and activation of their client proteins to regulate numerous intracellular signaling pathways. Previous studies demonstrated that pan-Hsp90 inhibitors reduce inflammatory signaling pathways resulting in a reduction of inflammation and pain but show toxicities in cancer-related clinical trials. Further, the role of Hsp90 isoforms in inflammation remains poorly understood. This study aimed to determine anti-inflammatory activities of Hsp90 isoforms selective inhibitors on the lipopolysaccharide (LPS)-induced inflammation in BV-2 cells, a murine microglial cell line. The production of inflammatory mediators such as nitric oxide (NO), interleukin 1 beta (IL-1β), and tumor necrosis factor-alpha (TNF-α) was measured. We also investigated the impact of Hsp90 isoform inhibitors on the activation of nuclear factor kappa B (NF-κB), nuclear factor erythroid 2–related factor 2 (Nrf2), and mitogen-activated protein kinases (MAPKs). We found that selective inhibitors of Hsp90β reduced the LPS-induced production of NO, IL-1β, and TNF-α via diminishing the activation of NF-κB and Extracellular signal-regulated kinases (ERK) MAPK. The Hsp90α, Grp94, TRAP1 inhibitors had limited effect on the production of inflammatory mediators. These findings suggest that Hsp90β is the key player in LPS-induced neuroinflammation. Thereby providing a more selective drug target for development of medications involved in pain management that can potentially contribute to the reduction of adverse side effects associated with Hsp90 pan inhibitors.

## Introduction

Neuroinflammation is characterized by a set inflammatory responses mediated by inflammatory mediators within the central nervous system (CNS) (DiSabato et al., 2016). Studies have demonstrated that neuroinflammation to contributes to the pathogenesis of several neurological disorders, such as pain, Alzheimer’s disease, Parkinson’s disease, and amyotrophic lateral sclerosis (Ji et al., 2016; Kwon and Koh, 2020; Ahmad et al., 2022). Microglial cells are resident macrophage-type cells and play a major role in neuroinflammatory (Graeber et al., 2011; Caraci et al., 2019; Kwon and Koh, 2020). Activation of microglial cells facilitate pain sensitization and other neurological disorders (Watkins et al., 2001; Ji et al., 2018; Tozaki-Saitoh and Tsuda, 2019). Neuroinflammation is also involved in the adverse effects associated with chronic treatment of opioid drugs (Eidson et al., 2017; Qu et al., 2017). Thus, understanding the regulation of neuroinflammation could provide evidence for improving management of neurological disorders.

Previous studies demonstrated that Hsp90 inhibition enhanced the antinociceptive effects of opioids via activating the ERK-RSK signaling pathway (Duron et al., 2020). This beneficial impact of Hsp90 inhibition could reduce the risk of side effects, such as tolerance and dependence, by reducing the opioid doses required for pain management. Hsp90 is a subclass of a family of chaperone proteins that assist in the folding of proteins to maintain structure and function (Streicher, 2019). Their expression is upregulated in response to cellular stress, diseases, or an increased need for protein synthesis to maintain cellular functioning (Sima and Richter, 2018). Hsp90 has gained interest as a novel target to develop medications for the treatment of cancer and neurodegenerative diseases (Costa et al., 2020). A key mechanism of Hsp90 inhibitors for cancer therapy is to regulate inflammatory responses via the regulation of secretory pathways, integrins, and toll-like receptor (TLR) signaling (Tsan and Gao, 2009). Numerous studies have examined the effects of Hsp90 on inflammatory signaling pathways in different models (Chatterjee and Burns, 2017; Dukay et al., 2019; He et al., 2019; Talaei et al., 2019). Upregulation of Hsp90 can activate ERK, JAK2, and STAT3, which increases the production of inflammatory mediators, such as IL-6, IL-1β, TNF-α, and NO (He et al., 2019). Thus, inhibition of Hsp90 has been an approach to discover drugs for treating diseases associated with inflammation (Chatterjee and Burns, 2017; Dukay et al., 2019; Talaei et al., 2019). Numerous non-selective Hsp90 inhibitors, including 17-N-allylamino-17-demethoxygeldanamycin (17-AAG), have been tested in preclinical models, and clinical trials and have shown promising effects in different disease states (Talaei et al., 2019). However, hepatic, cardiac, and ocular toxicities associated with Hsp90 pan inhibition have been found in clinical trials (Talaei et al., 2019; Miles et al., 2020). Therefore, discovering Hsp90 inhibitors with fewer adverse effects would facilitate their application in human patients.

There are four isoforms in the Hsp90 family: Hsp90α, Hsp90β, GRP94, and TRAP1. Hsp90α and Hsp90β are the major isoforms, expressed predominantly within the cytoplasm, while GRP94 is expressed in the endoplasmic reticulum and TRAP1 in the mitochondria (Hoter et al., 2018; Lei et al., 2019). Each isoform of Hsp90 has different client proteins, although they share a high rate of similarity on structure (Hoter et al., 2018). Targeting specific Hsp90 isoforms could be a strategy to reduce or eliminate off-target interactions.

This study aims to examine the activities of novel Hsp90 isoform selective inhibitors on the LPS-induced inflammatory responses in microglial cells. We tested the impact of selective inhibitors of Hsp90α, Hsp90β, GRP94, and TRAP-1 on LPS-induced NO and cytokine production, and activation of NF-κB, Nrf2, and MAPKs. The findings from this study provide a better understanding of the functional role of each isoform of Hsp90 on inflammatory responses.

## Materials and methods

### Drugs and reagents

The inhibitors for specific Hsp90 isoforms [KUNA115 and NDNA1065 (Hsp90α), KUNB106 and its analog, NDNB1151 (Hsp90β), KUNG65 (Grp94), T1, T2, and T3 (TRAP1)] were synthesized and purified by the laboratory of Dr. Brian Blagg at the University of Notre Dame [(Crowley et al., 2017; Mishra et al., 2021a; Mishra et al., 2021b; Merfeld et al., 2023), Figure 1]. Lipopolysaccharide (LPS, from *E. coli* O111:B4), 17-AAG and penicillin:streptomycin (tissue culture) were purchased from VWR (Radnor, PA, United States). Dulbecco’s Modified Eagle Medium (DMEM), trypsin-EDTA, fetal bovine serum (FBS), and G418 disulfate solution were obtained from Corning (Corning, NY, United States). Griess reagents for nitric oxide assay (sodium nitrite, sulfanilamide, N-1-naphthylethylenediamine) were purchased from Sigma-Aldrich (St. Louis, MO, United States). Antibodies used for Western blots include goat anti-rabbit IgG-horseradish peroxidase, and goat anti-mouse IgG-horseradish peroxidase (Invitrogen, Waltham, MA, United States); p-ERK, t-ERK, p-p38, t-p38, p-JNK, and t-JNK (Cell Signaling, Beverly, MA, United States).

**FIGURE 1:**
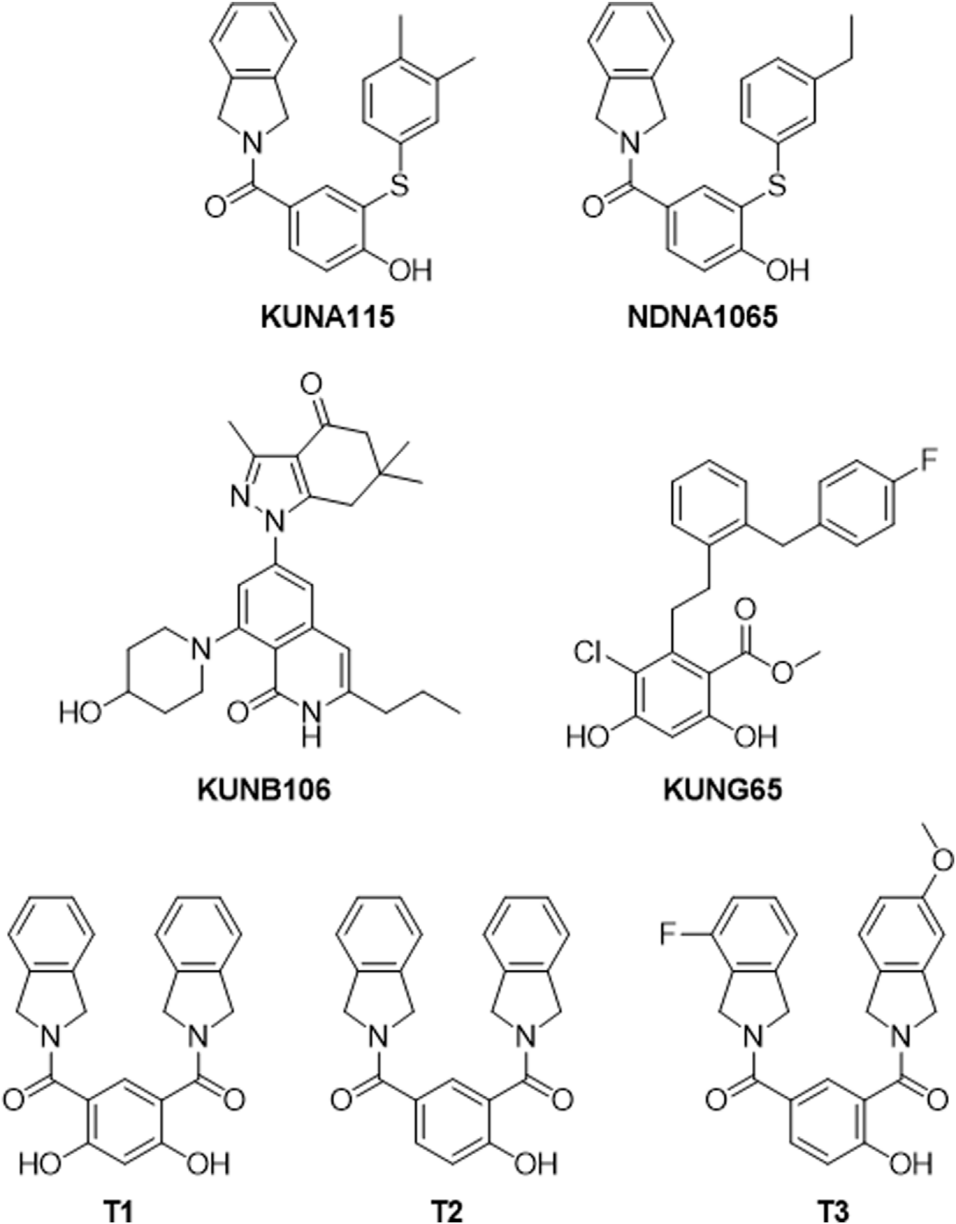
Structures of Hsp90 isoform selective inhibitors. KUNA115 and NDNA1065 are Hsp90α (cytosolic and inducible Hsp90 isoform) selective inhibitors. KUNB106 is a molecule selectively binds to Hsp90β (cytosolic isoform) N-terminal site and induces the degradation of Hsp90β-dependent client proteins. KUNG65 selectively inhibits the activity of Grp94, the endoplasmic reticulum resident isoform of Hsp90. T1, T2, and T3 are small molecules that bind to the N-terminal of TRAP1 (mitochondrial isoform of Hsp90) resulting in interrupting TRAP1 function.

### Cell lines and cell culture

The murine microglial BV-2 cells stably transfected with nuclear factor-κB (NF-κB) or Nrf2 reporter were generously provided by Dr. Grace Sun and Dr. Valeri Mossine from University of Missouri-Columbia. Cells were maintained in DMEM, supplemented with 5% fetal bovine serum (FBS), 1% penicillin/streptomycin, and G418. They were cultured at with 95% air and 5% CO₂ at 37°C.

### Nitric oxide assay

Nitric oxide (NO) concentration was measured using Griess reagents as described by Lei et al. (2015). The cells (1 × 10^5^ cells/well) were seeded into a 96-well plate and cultured overnight to reach >95% of confluence. Cells were starved in the serum-free DMEM for 1 h, and then treated with different Hsp90 inhibitors for 1 h prior to the co-treatment with LPS for 24 h. Sodium nitrite (NaNO₂) dilutions were prepared to establish a standard curve. Fifty microliters of cell culture medium were transferred into a 96-well ELISA plate and mixed with an equivalent amount of Griess reagents (7.5 mM sulfanilamide, 0.75 M HCl, and 7.5 mM naphthyl ethylenediamine). After incubation for 10 min at room temperature, the absorbance was measured at 548 nm using a mini-plate reader (Biotek, Winooski, VT, United States). The concentrations of nitrite were calculated based on a sodium nitrite standard curve.

### Quantitative polymerase chain reaction (qPCR)

In order to investigate the production of the proinflammatory mediators TNF-α and IL-1β, qPCR was performed. BV-2 cells were treated with 17-AAG or KUNB10 and LPS for 3 h as described in Western blot. Cells were collected after washing with ice-cold PBS. Total ribonucleic acid (RNA) was isolated using the RNeasy Mini Kit (Qiagen, Germantown, MD). The concentrations and quality of RNA were detected by the NanoDrop Spectrophotometer (Fisher Scientific, Waltham, MA). Total RNA (1 μg) was mixed with iScript Reverse Transcription reagents (BioRad, Hercules, California) to synthesize complimentary deoxyribonucleic acid (DNA). The qPCR was performed to determine gene expression using Universal SYBR Green Supermix (BioRad, Hercules, California) in the c1000Touch Thermal Cycler/CFX96 Touch Real-Time PCR Detection System (BioRad, Hercules, California). The expression of GAPDH was used as reference for calculating the expression of TNF-α and IL-1β using the ^ΔΔ^Ct relative expression method (Livak and Schmittgen, 2001). The sequences of primers were TNF-α F: 5′-CTC TTC AAG GGA CAA GGC TG-3′, R: 5′-TGG AAG ACT CCT CCC AGG TA-3’; IL-1β F: 5′- ATG CCT TCC CCA GGG CAT GT -3′, R: 5′-CTG AGC GAC CTG TCT TGG CCG-3′; GAPDH F: 5′-TCC TGC ACC ACC AAC TGC TTA G-3′, R: 5′-GAT GAC CTT GCC CAC AGC CTT G-3’.

### Western blot

Cells were plated in a 12-well plate and cultured overnight to reach >95% confluence. After starving for 1 h in the serum free DMEM, cells were pretreated with 17-AAG or Hsp90 isoform selective inhibitors (1 or 10 μM) for 1 h, followed by co-treatment with 1 μg/mL LPS for another 15 min to evaluate the phosphor- and total-ERK, JNK, and p38. After treatment, cells were washed with ice-cold PBS and lysed by adding 50 μL of lysed in radioimmunoprecipitation assay buffer (RIPA) with protease inhibitor (Pierce Biotechnology, Rockford, IL) and phosphatase inhibitors (ThermoFisher Scientific, Inc., Waltham, MA). Cell lysates were transferred into a new 1.5 mL tube, sheared with 26-gauge needle, centrifuged at 14,000 g at 4°C for 10 min, and the supernatants were stored at −80°C freezer. The protein was run on precast Tris-glycine gels and transferred to nitrocellulose membrane (Bio-Rad Laboratories, Hercules, CA). The blots were blocked with 5% nonfat milk in tris-buffered saline (TBS) and incubated with primary antibody (1:1,000 dilution) in 5% bovine serum albumin (BAS) in TBS containing 0.1% Tween-20 (TBST) overnight at 4°C. The blots were incubated with goat anti-rabbit or goat anti-mouse IgG-horseradish peroxidase conjugated antibodies (1:5,000 dilution) in 5% milk in TBST for 1 h at room temperature. The antibody-antigen complexes were detected using enhanced chemiluminescence detection kit (Pierce Biotechnology, Rockford, IL) and imaged with a GeneSys imaging system. All image bands were quantified using Scion Image. The expression of p-ERK, p-JNK, or p-p38 were normalized to the t-ERK, t-JNK, or t-p38, respectively. The normalized intensities were further normalized to a positive control present on the same blot.

### Luciferase assay

The effect of Hsp90 inhibitors on LPS-induced activation of NF-κB and Nrf2 signaling pathway was detected using a luciferase assay. BV-2 cells transfected with NF-κB or Nrf2 reporter were seeded in a 96-well plate and cultured overnight. The cells were starved in the serum free DMEM for 1 h and treated with Hsp90 inhibitors for 1 h prior to the co-treatment with LPS for 3 h. Cells were lysed using the luciferase lysis buffer, and equivalent of luciferase substrates were added. The luminescence was detected immediately by a mini-plate reader.

### Statistical analysis

All data represents the Mean ± standard error of mean (SEM) from at least three independent experiments. All treatment effects were analyzed by one-way ANOVA and Tukey’s multiple comparison. All graphing and statistical analyses were performed using GraphPad Prism 8.3 (San Diego, CA). A *p* < 0.05 was considered to indicate statistical significance.

## Results

### Effect of specific isoform Hsp90 inhibitors on LPS-induced NO production

**FIGURE 2:**
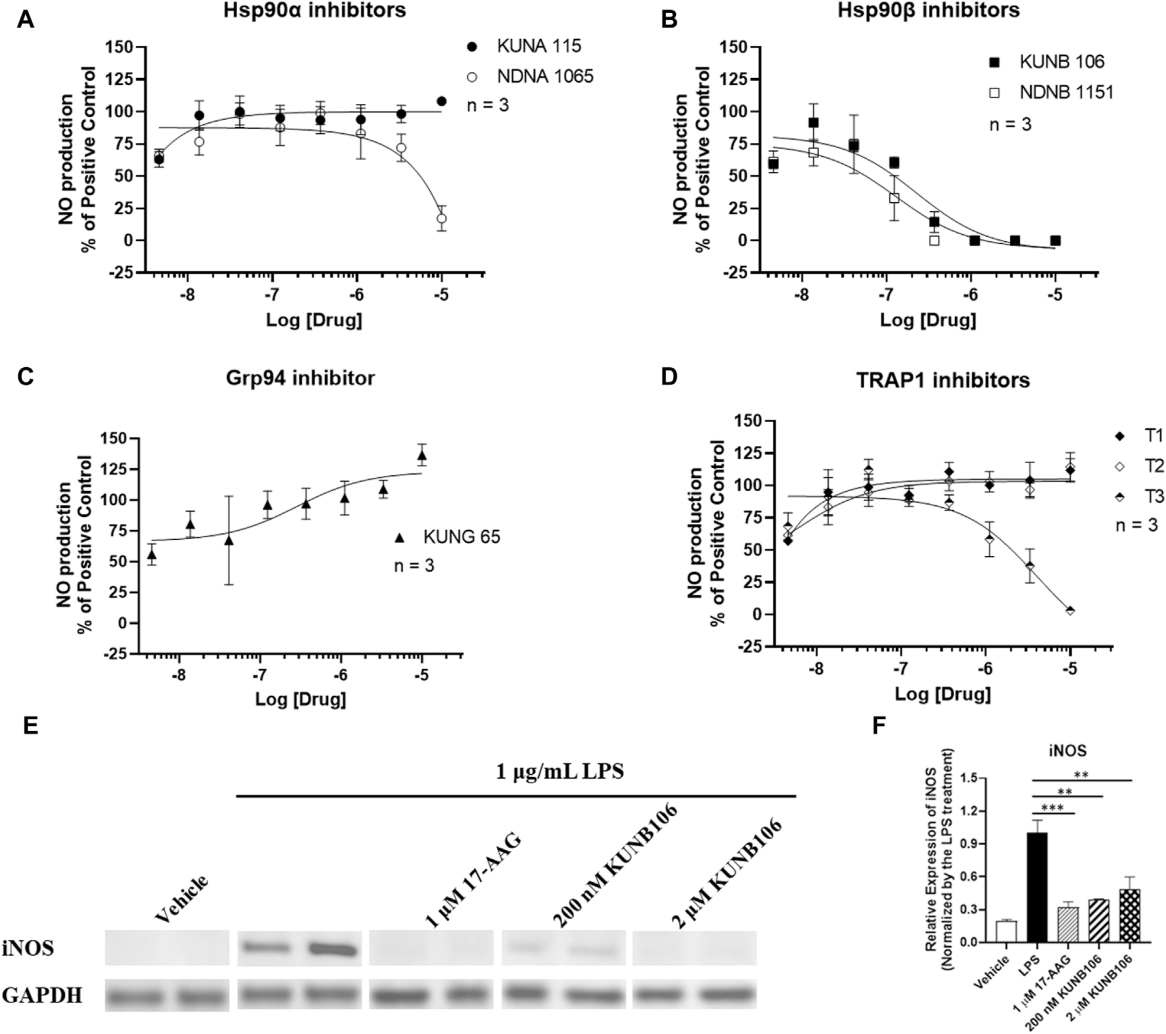
Impact of Hsp90 isoform inhibitors on LPS-induced nitric oxide (NO) production in BV-2 microglial cells. BV-2 microglial cells were seeded in 96-well plates and cultured in DMEM with 5% FBS and 1% P/S overnight. Cells were serum starved in DMEM without FBS for 1 h. Cells were pre-treated with selective inhibitors for Hsp90α **(A)**, Hsp90β **(B)**, Grp94 **(C)**, or TRAP1 **(D)** for an hour, followed by stimulation with 1 μg/mL LPS for 24 h. Cell culture medium was harvested for NO measurement using Griess’ Reagents. The cell lysates from the treatment with 17-AAG and KUNB106 were harvested, and the protein (∼20 µg) was run on 4%–15% bis-tris gels and transferred to a nitrocellulose membrane. The blots were blocked by 5% nonfat milk at room temperature (RT), then incubated with iNOS and GAPDH antibody overnight at 4°C. After washing, the blots were incubated with secondary antibody at RT for 1 h. Western blot analysis showing a representative experiment **(E)**, and the bar graphs **(F)** representing ratios of iNOS and GAPDH. Data from cells treated LPS alone were considered as 100%, and all the other data were normalized by LPS group. Data from 3 independent experiments are presented as Mean ± SEM. ** and ***, *p* < 0.01 and 0.001 versus the LPS treatment group by one-way ANOVA with Tukey *post hoc* test.

Treatment of LPS promoted the production of NO (5.114 ± 0.813 nM vs. not detectable NO in vehicle control group), and Hsp90 inhibitors exhibited various effects on the LPS-induced NO production in BV-2 cells (Figure 2). Treatment with 17-AAG produced a dose-dependent reduction in NO production (Supplementary Figure S1) as documented in previous studies (Miao et al., 2008; Petersen et al., 2012; Todurga Seven et al., 2022). Both Hsp90β inhibitors (KUNB106 and NDNB1151) and a TRAP1 inhibitor (T3) inhibited the production of NO induced by LPS in a dose-dependent matter (Figure 2). Hsp90α inhibitors (KUNA115 and NDNA1065) showed no or very limited effect on LPS-induced NO production (Figure 2). Grp94 inhibitor (KUNG65) and two TRAP1 inhibitors (T1 and T2) had no impact on the NO production. All Hsp90 isoform inhibitors exhibited a similar effect on IFNγ-induced NO production as shown in the LPS-stimulated BV-2 cells (Supplementary Figure S2). Further, we found that treatment with KUNB106 reduced the expression of inducible nitric oxide synthase (Figures 2E, F), the enzyme synthesizing NO in immune cells.

### Effect of Hsp90 inhibitors on the production of pro-inflammatory cytokines

**FIGURE 3:**
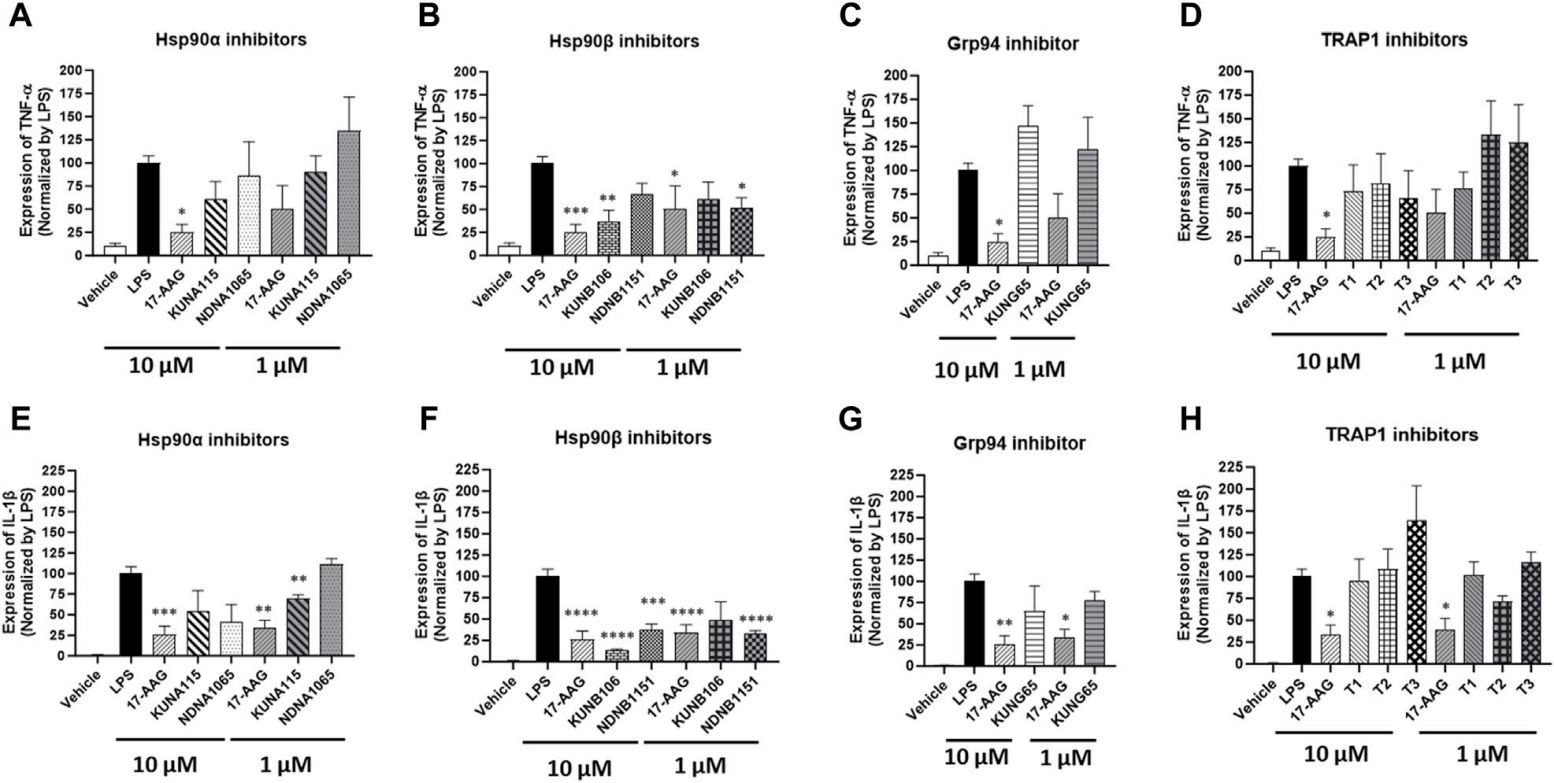
Impact of Hsp90 isoform selective inhibitors on LPS-induced TNF-α and IL-β expression in BV-2 cells. BV-2 cells were seeded into the 6-well plates and cultured overnight in DMEM/5%FBS. Cells were treated with Hsp90 inhibitors for 1 h followed by co-treatment with LPS (1 μg/mL) in serum free DMEM for 3 h. The cell pellets were harvested for total RNA extraction, and the cDNA was synthesized using iScript™ Reverse Transcription Supermix. The expression of GAPDH, TNF-α **(A–D)**, and IL-1β **(E–H)** was measured using iQ™ SYBR^®^ Green Supermix. The treatment with LPS was used as a positive control. All the data were normalized by the LPS in each experiment and presented as mean ± SEM. *, **, ***, and ****, *p* < 0.05, 0.01, 0.001, and 0.0001 versus the LPS treatment group by one-way ANOVA with Tukey *post hoc* test.

The expression of IL-1β and TNF-α was increased in the LPS-stimulated BV-2 cells, and some Hsp90 isoform selective inhibitors were able to reduce the expression of those inflammatory cytokines (Figure 3). KUNA115 and NDNA1065, the Hsp90α selective inhibitors, had a modest effect on the expression of IL-1β and TNF-α (Figures 3A,E). KUNA115 and NDNA1065 (at the 10 µM concentration) reduced the production of IL-1β by 45% and 59%, respectively, but no effect on TNF-α production. KUNB106 and NDNB1151, the Hsp90β selective inhibitors, significantly reduced the expression of IL-1β and TNF-α (Figures 3B,F). KUNB106 (10 µM) attenuated 86% and 63% of LPS-induced IL-1β and TNF-α, while NANB1151 (both 1 μM and 10 µM) reduced up to 68% of those cytokines. The selective inhibitors for Grp94 (i.e., KUNG65) and TRAP1 (T1, T2, and T3) had limited effect on the production of TNF-α and IL-1β, which the exception of slight stimulatory effect of T3 on IL-1β (Figure 3C; 3D; 3G; 3H).

### Effect of Hsp90 inhibitors on the activation of NF-κB and Nrf2 signaling pathways

**FIGURE 4:**
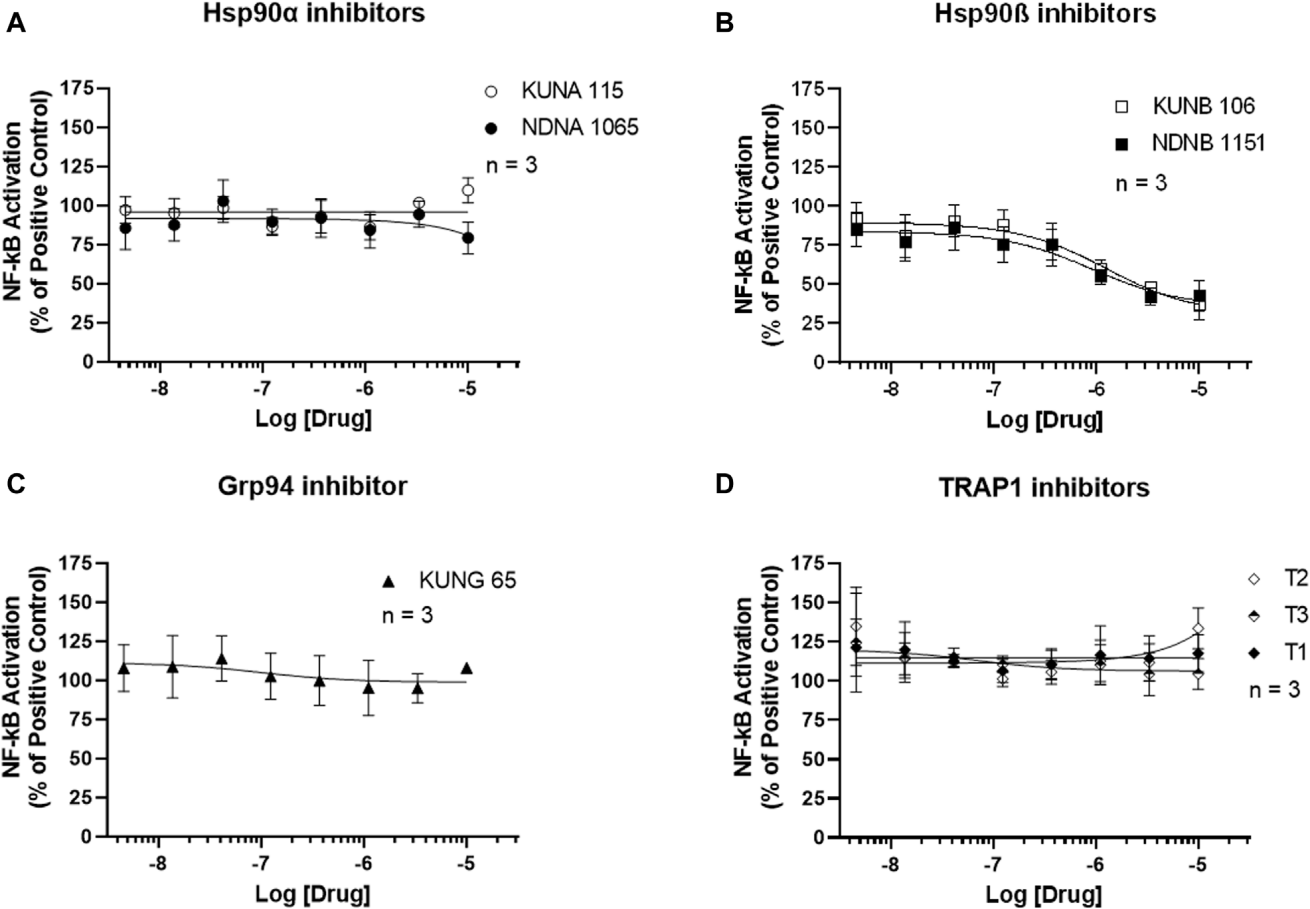
Selective inhibitors for Hsp90β reduces the NF-κB activation in the LPS-treated BV-2 cells. **(A–D)** BV-2 cells stably express NF-κB reporter were seeded in 96-well plates and cultured overnight. After serum starve for 1 h, cells were pre-treated with Hsp90 inhibitors for an hour, followed by stimulation with 1 μg/mL LPS for 3 h. Cell culture medium was removed, and 60 uL of luciferase lysis buffer was added to each well. After adding the luciferase substrates, the luminescent signal was read in a mini-plate reader immediately. Data from cells treated LPS alone were considered as 100%, and all the other data were normalized by LPS group. Data from 3 independent experiments are presented as Mean ± SEM.

NF-κB activation was measured using the Luciferase Assay. As shown in Figure 4, selective inhibitors for Hsp90α, Grp94, and TRAP1 had no effect on the activation of NF-κB in the LPS-stimulated BV-2 cells. Both Hsp90β inhibitors, KUNA115 and NDNA1065, dose-dependently reduced the LPS- or IFNγ-induced NF-κB activation (Figure 4B; Supplementary Figure S3).

**FIGURE 5:**
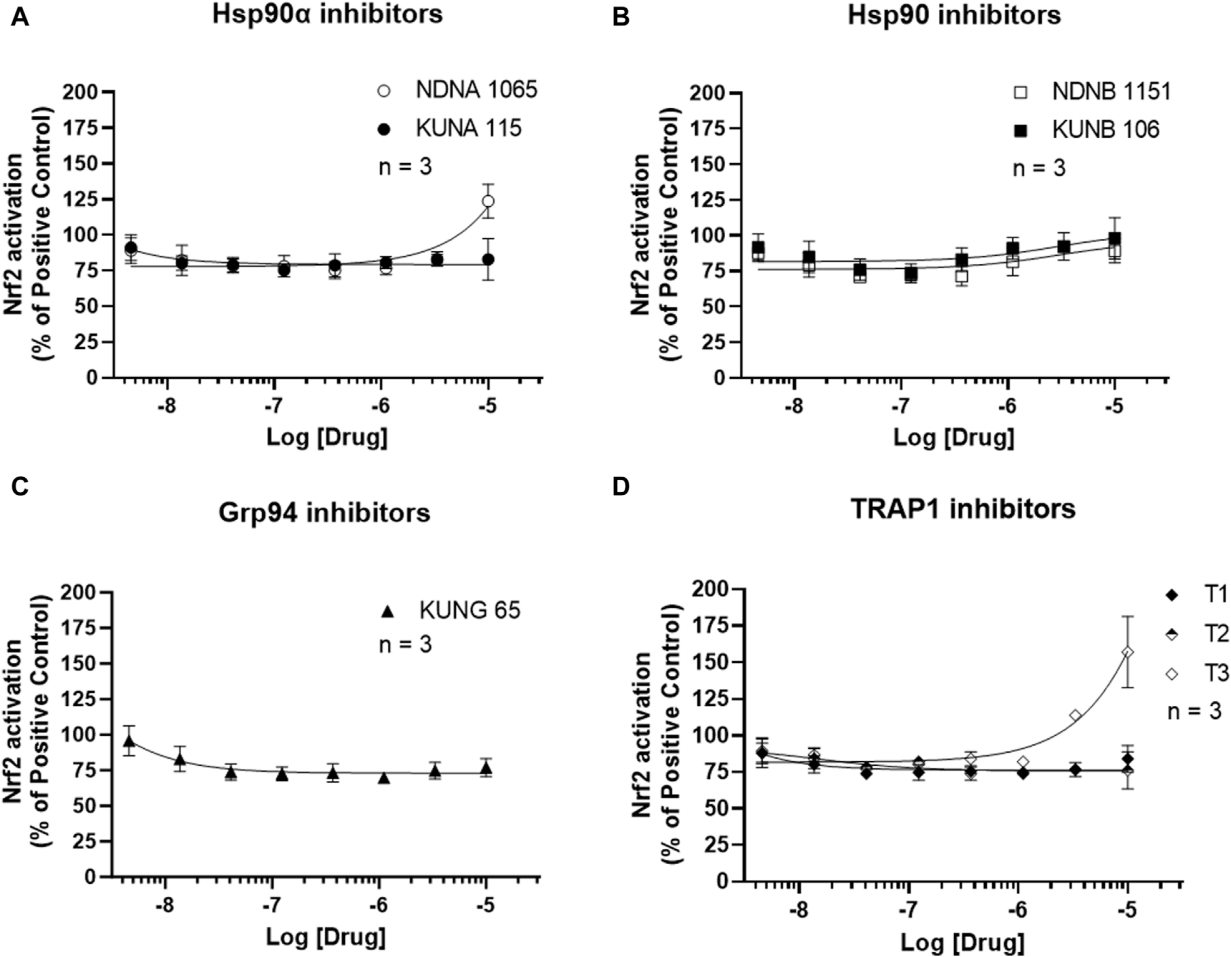
Effect of Hsp90 inhibitors on the activation of Nrf2 in BV-2 cells. **(A–D)** BV-2 cells stably express ARE reporter were seeded in 96-well plates and cultured overnight. After serum starving for 1 h, cells were treated with Hsp90 inhibitors for 3 h. Cells were lysed by adding luciferase lysis buffer, and equal amount of luciferase substrates were added. The luminescent signal was read in a mini-plate reader immediately. Data from untreated cells were used as reference to normalize the data from other groups. Data from 4 independent experiments are presented as Mean ± SEM.

Activation of the antioxidant pathway, Nrf2, exhibits anti-inflammatory activities (Ali et al., 2020). The impact of Hsp90 isoform selective inhibitors on Nrf2 activation is presented in Figure 5 and Supplementary Figure S4. Treatment with NDNA1065 (a Hsp90α inhibitor) and T3 (a TRAP2 inhibitor) increased the Nrf2 activation in the LPS- or IFNγ-treated BV-2 cells. All the other Hsp90 isoform inhibitors had no effect on Nrf2 signaling.

### Effect of Hsp90 inhibitors on the activation of MAPKs

**FIGURE 6:**
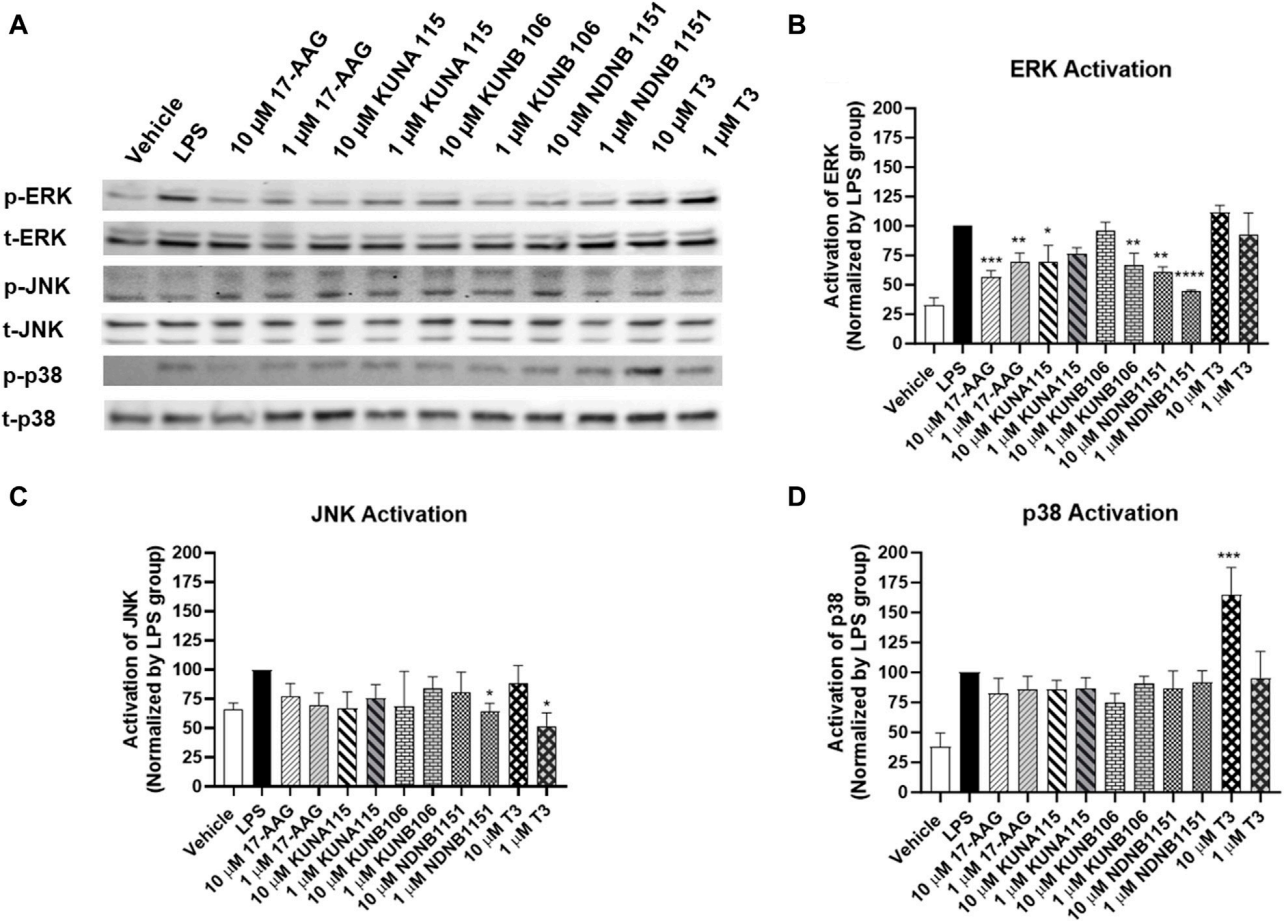
Hsp90β inhibitors decrease activation of ERK. BV-2 cells were treated with Hsp90 inhibitors for 1 h followed by co-treatment with LPS (1 μg/mL) in serum free DMEM for 15 min. The cells were lysed using RIPA buffer. The protein (∼20 µg) was run on 4%–15% bis-tris gels and transferred to a nitrocellulose membrane. The blots were blocked by 5% nonfat milk at room temperature (RT), then incubated with phosphorylated and total ERK, JNK, or p38 antibodies overnight at 4°C. After washing, the blots were incubated with secondary antibody at RT for 1 h. Western blot analysis showing a representative experiment **(A)**, and the bar graphs **(B–D)** representing ratios of phosphorylated and total proteins using LPS as control (100%). Results from at least 3 independent experiments are expressed as the mean ± SEM. *, **, ***, and ****, *p* < 0.05, 0.01, 0.001, and 0.0001 versus the LPS treatment group by one-way ANOVA with Tukey *post hoc* test.

According to the effect of Hsp90 isoform inhibitors on the production of NO, IL-1β, and TNF-α, we selected several Hsp90 isoform inhibitors to evaluate MAPKs activation (Figure 6). Exposure to KUNA115, a Hsp90α selective inhibitor, only had modest but statistically significant reduction on phosphorylation of ERK, but not p38 and JNK in the LPS-stimulated BV-2 cells. Similar to KUNA115, Hsp90β inhibitors significantly reduced the phosphorylation of ERK by up to 56% and slightly reduced the phosphorylation of JNK. T3, a TRAP1 selective inhibitor, reduced the phosphorylation of JNK by 48% but increased the phosphorylation of p38 by 65%.

## Discussion

In this study, we demonstrate that Hsp90β is the major isoform of Hsp90 involved in the production of inflammatory mediators (i.e., NO, IL-1β, and TNF-α) in the LPS- or IFNγ-stimulated BV-2 microglial cells, through activation of NF-κB and ERK MAPK. Due to the well documented relationship between Hsp90 and inflammation, our study aimed to determine which isoforms or co-chaperones were involved in this activity.

Previous studies demonstrated that Hsp90 inhibition could reduce the inflammation via various molecular mechanisms including modulation of inflammatory signaling pathways and reduction in inflammatory mediator production (Barabutis et al., 2019; He et al., 2019; Choudhury et al., 2020; Shi et al., 2020; Zhang et al., 2020; Nizami et al., 2021; Zhang et al., 2021). Hsp90 inhibitors (i.e., EC144, geldanamycin, and 17-DMAG, a 17-AAG derivative) diminished the activation of NLRP3 (NACHT, LRR and PYD domain-containing protein 3) signaling pathway to reduce the production of IL-1β, IL-6, IL-18, in the cell and animal inflammatory models as well as the human samples (Choudhury et al., 2020; Shi et al., 2020; Nizami et al., 2021). Hsp90 inhibitor 17-DMAG can also reduce the p53 phosphorylation, leading to reduction in IL-2 and IL-10 in LPS-induced inflammation in bovine pulmonary arterial endothelial cells and in lungs of mouse models (Barabutis et al., 2019). In addition, Hsp90 inhibition can reduce the pro-inflammatory responses by inhibiting NF-κB, MAPK, and JAK2-STAT3 pathways in M1 macrophages (Zhang et al., 2021). Geldanamycin, a Hsp90 inhibitor, decreased the toll-like receptor 4 (TLR4) and NF-κB signaling in the myocardial inflammation (Zhang et al., 2020; Zhang et al., 2021). However, the role of Hsp90 isoform on the inflammatory responses is still unclear since most of those studies have used the Hsp90 pan inhibitors which universally block the function of Hsp90. By using KUNB31, a Hsp90β selective inhibitor, Nizami and colleagues found that Hsp90β had no effect on the LPS-induced inflammation in bone marrow-derived macrophages and in the mouse model (Nizami et al., 2021). However, another study reported that inhibiting Hsp90β by siRNA attenuated the heat shock-induced inflammatory responses in the N9 microglial cells (He et al., 2019), which agrees with the findings from our study.

The present study applied multiple Hsp90 isoform selective inhibitors to the inflammatory condition induced by LPS in BV-2 microglial cells. Hsp90α and TRAP1 selective inhibitors showed some anti-inflammatory activities which may be contributed by activating the Nrf2 signaling pathway. However, Hsp90β inhibitors consistently exhibited activities to reduce the production of NO (by inhibiting the expression of iNOS), IL-1β, and TNF-α via diminishing the activation of NF-κB and ERK signaling pathways but had no effect on Nrf2 activation. The difference of Hsp90 isoform inhibitors on the inflammatory responses may be contributed by the cellular locations, client proteins, and biological functions of each isoform of Hsp90 (Hoter et al., 2018). We also found that Hsp90 inhibitors (KUNA115, KUNB106, and NDNB1151) had greater effect on reducing the expression of IL-1β compared to that on TNF-α expression. Previous studies have demonstrated that Hsp90 ATPase activity is essential for the priming and activation of NLRP3 and NLRC4 inflammasome which is required for the expression of IL-1β, but not for TNF-α (Zuo et al., 2018; Shi et al., 2020). Thus, inhibiting Hsp90 activity could exhibit a stronger inhibition on expression of IL-1β via targeting multiple intracellular signaling pathways. Overall, these findings indicate that Hsp90β is the main isoform involved in the LPS-induced inflammation in BV-2 microglial cells. In this study, three TRAP1 inhibitors (T1, T2, and T3) were tested, however, only T3 showed some anti-inflammatory activities. A previous study reported that T3 had higher selectivity to TRAP1 compared to the other two molecules (Merfeld et al., 2023), which could contribute to the different effects of T1, T2, and T3 on the inflammatory responses. More studies are needed to determine the mechanism responsible for such differences.

Neuroinflammation is a critical response to protect the central nervous system, and is also involved in the pathological development of neurological disorders, such as neurodegenerative diseases and pain signaling (Liu et al., 2020; Rauf et al., 2022). The glial cells, including microglial and astrocytes, are mainly involved in the initiation of neuroinflammation within the central nervous system. Upon the stimulation, microglial cells and astrocytes increase the production of numerous proinflammatory mediators, such as IL-1β, IL-6, TNF-α, and NO. Our previous studies found that Hsp90 inhibitors, such as 17-AAG and KU-32, could regulate the opioid analgesic effect and reduce the opioid tolerance (unpublished data) in different pain models via ERK signaling pathway (Lei et al., 2017; Duron et al., 2020; Stine et al., 2020). This study further confirms that ERK signaling pathway is important for the medical benefit of Hsp90 inhibitors. As discussed above, selective inhibitors for Hsp90β, which is the main isoform responding to LPS-induced inflammation, reduce the activation of ERK by up to 55%. Combined with the reduction in NF-κB, Hsp90β inhibitors are able to significantly inhibit the production of NO, IL-1β, and TNF-α in the activated microglial cells. Neuroinflammation is an important component in pain sensitization (Watkins et al., 2001; Ji et al., 2018; Tozaki-Saitoh and Tsuda, 2019). Previous studies also demonstrated that neuroinflammation contributes to the development of morphine tolerance (Eidson et al., 2017; Qu et al., 2017). Therefore, reducing neuroinflammation has been an approach for improving pain management and may reduce the risk of opioid tolerance.

This study was also limited in a few ways. First, the study was only performed in one type of glial cells. It is possible that the Hsp90 isoform inhibitors may have different effects in different types of glial cells or other immune cells which contribute to the development of neurological disorders. The second limitation was that the findings were not verified in animal models. As indicated in our previous study (Lei et al., 2017), the results discovered in the cell models had limited representation to the animal models. Third, this study does not evaluate the misfolded proteins after treatment with Hsp90 inhibitors. Hsp90 is a molecular chaperone protein and is involved in the folding, maturation, and activities of more than 400 proteins (Chaudhury et al., 2021). Inhibition or deletion of Hsp90 can cause accumulation of misfolded proteins which could promote the inflammatory responses (Finka et al., 2011). Further, it is still unclear how Hsp90β inhibitors reduce the activation of NF-κB and ERK signaling pathways. Numerous proteins play essential activities in NF-kB and MAPK signaling pathways (both upstream and downstream) (Roberts et al., 2021). It would be interesting to verify the impact of the tested Hsp90 inhibitors on those proteins associated with the NF-kB and MAPK signaling in our future experiments. In addition, the impact of Hsp90 isoform inhibitors were not tested in disease conditions, such as pain and morphine tolerance. Most recently, a study reported that spinal administration of inhibitors of Hsp90α, Hsp90β, and Grp94 could reduce the opioid tolerance (Duron et al., 2021). Overall, further studies are needed to verify the beneficial activities of Hsp90 isoform selective inhibitors and to evaluate their clinical implications.

## Conclusion

Identifying the isoforms and components that account for the anti-inflammatory effects of Hsp90 inhibition provides insight to the mechanisms of signaling pathways. The findings from this project suggest that Hsp90β is a more specific drug target for developing medications related to Hsp90, which could reduce side effects associated with Hsp90 nonselective inhibitors. However, additional testing is needed to provide a better understanding of Hsp90β on neuroinflammation.

## Data Availability

The original contributions presented in the study are included in the article/Supplementary Material, further inquiries can be directed to the corresponding author.

## Supplementary material

SUPPLEMENTAL FIGURE S1 Impact of 17-AAG on NO production and NF-κB activation in BV-2 cells treated with LPS or IFN-γ. Treatment with 17-AAG, a Hsp90 pan inhibitor, dose-dependently reduces the NO production and NF-κB activation induced by LPS **(A,B)** or IFN-γ **(C,D)** in the BV-2 microglial cells. Data from cells treated LPS or IFN-γ alone were considered as 100%, and all the other data were normalized by LPS or IFN-γ group in the same experiment. Data from 3 independent experiments are presented as Mean ± SEM.

SUPPLEMENTAL FIGURE S2 Impact of Hsp90 inhibitors on IFN-γ-induced NO production in BV-2 cells. The Hsp90β selective inhibitors (KUNB 106 and NDNB 1151) and a TRAP1 selective inhibitor (T3) reduce the NO production induced by IFN-γ in a dose-dependent manner. All the data are normalized by the data from cells treated IFN-γ alone and presented as Mean ± SEM (n = 4).

SUPPLEMENTAL FIGURE S3 Impact of Hsp90 inhibitors on IFN-γ-induced NF-κB activation in BV-2 cells. Hsp90β selective inhibitors, but not any other Hsp90 isoform inhibitors, partially reduces the IFN-γ-induced NF-κB activation in BV-2 microglial cells. All the data are normalized by the data from cells treated IFN-γ alone and presented as Mean ± SEM (n = 4).

SUPPLEMENTAL FIGURE S4 Impact of Hsp90 inhibitors IFN-γ-induced Nrf2 activation in BV-2 cells. Treatment with T3, TRAP1 selective inhibitor, modestly enhances the activation of Nrf2 in the BV-2 microglial cells treated with IFN-γ **(D)**. Data from cells treated IFN-γ alone were considered as 100%, and all the other data were normalized by IFN-γ group. Data from 4 independent experiments are presented as Mean ± SEM.
