# Supplementary figures and images for: Anti-inflammatory activities of novel heat shock protein 90 isoform selective inhibitors in BV-2 microglial cells
Source: Front Mol Biosci. 2024 May 2;11:1405339. doi: 10.3389/fmolb.2024.1405339 (PMC11096514; doi:10.3389/fmolb.2024.1405339)

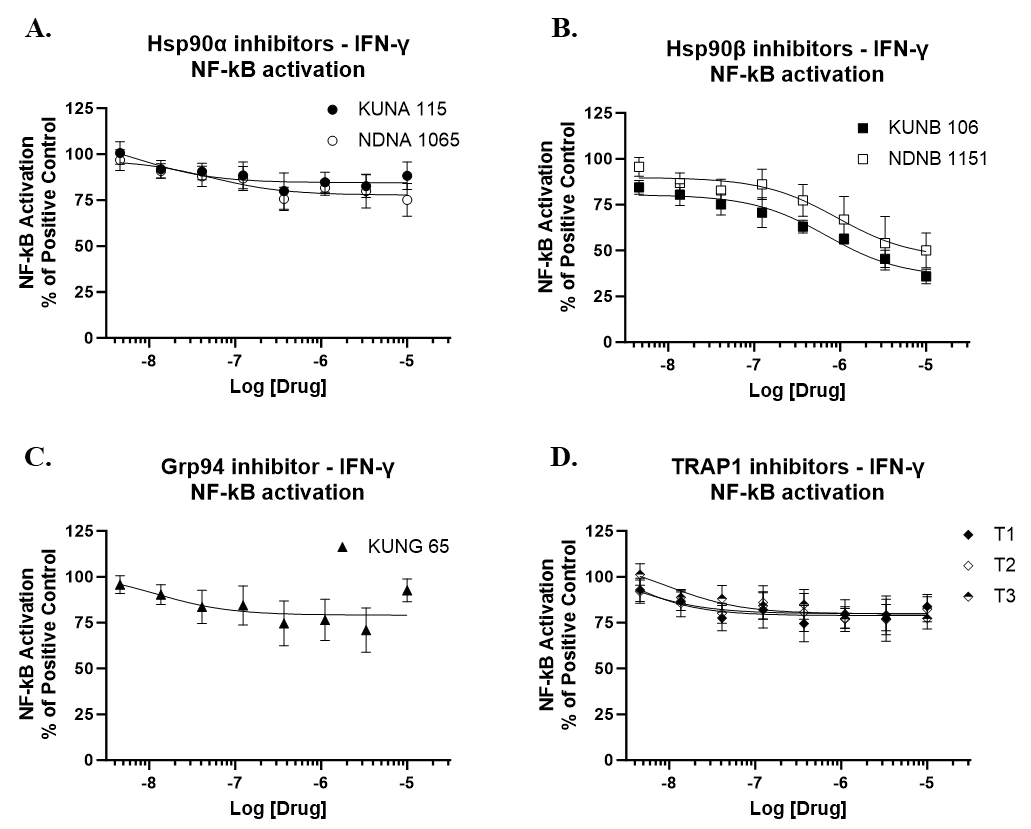

Supplement: Supplementary file 1 [file Image3.tif]

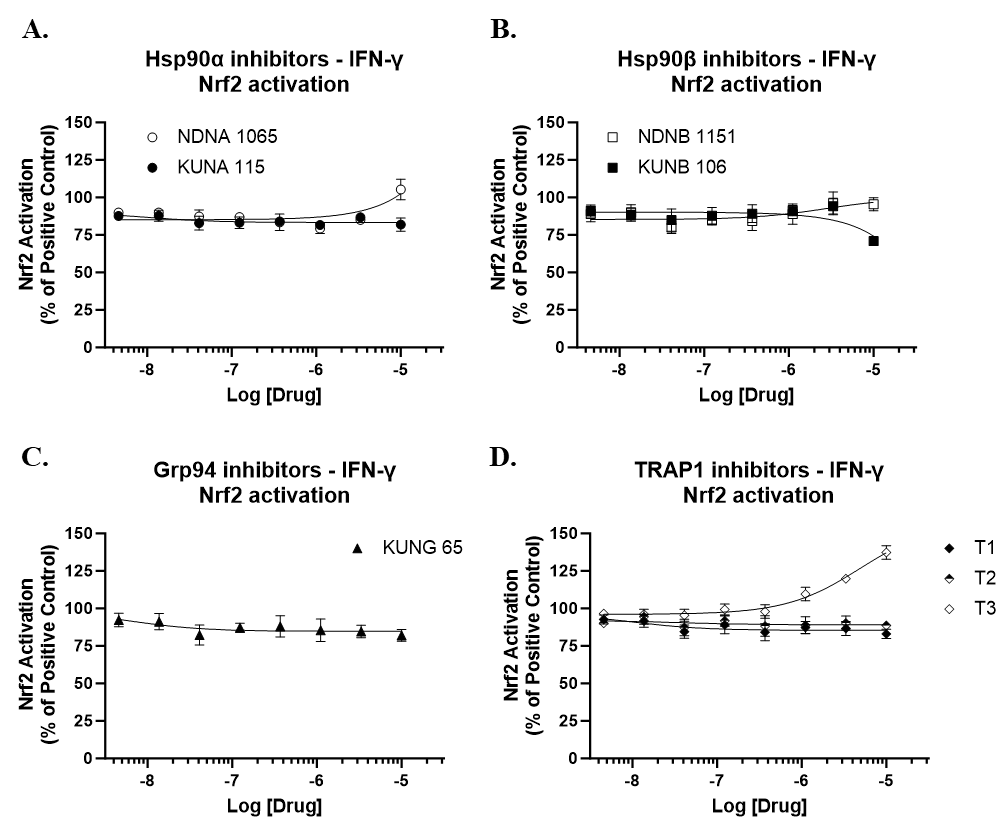

Supplement: Supplementary file 2 [file Image4.tif]

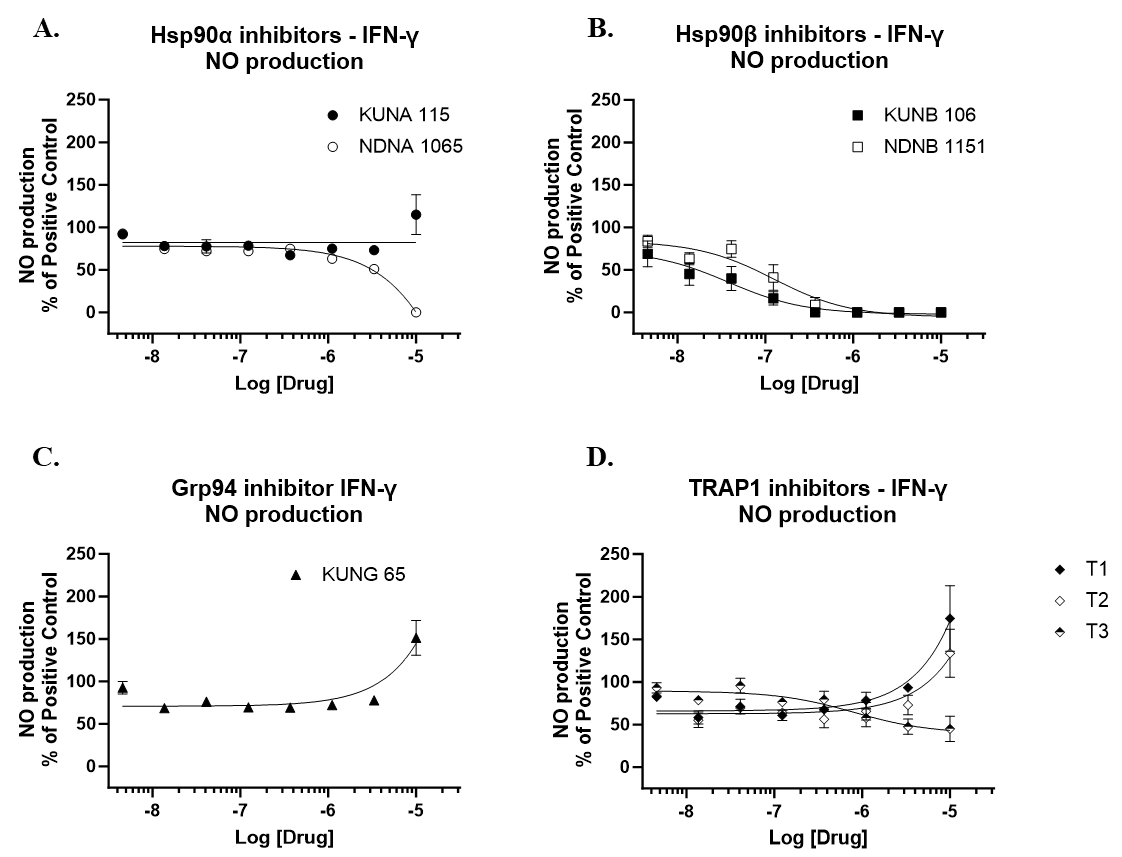

Supplement: Supplementary file 3 [file Image2.tif]

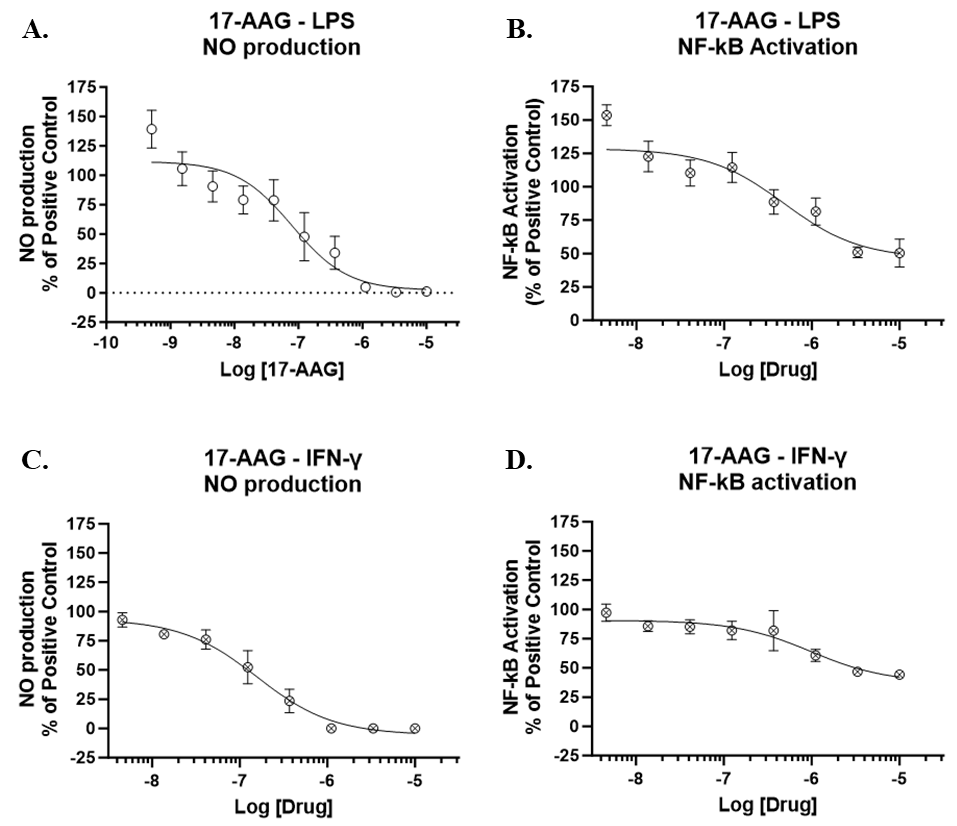

Supplement: Supplementary file 4 [file Image1.tif]
